# Editorial: Stem cell and translational medicine research in endocrine diseases

**DOI:** 10.3389/fendo.2024.1483050

**Published:** 2024-09-09

**Authors:** Wei Wei

**Affiliations:** ^1^ Center for Regeneration and Aging Medicine, International School of Medicine, the Fourth Affiliated Hospital, International Institutes of Medicine, Zhejiang University, Yiwu, Zhejiang, China; ^2^ Zhejiang Key Laboratory of Precision Diagnosis and Treatment for Lung Cancer, the Fourth Affiliated Hospital, Zhejiang University School of Medicine, Yiwu, Zhejiang, China

**Keywords:** stem cell (SC), translational medicine (TM), endocrine diseases, diabetes mellitus, tissue regeneration

This Research Topic focuses on the recent advances in stem cell and translational medicine research in endocrine diseases. Endocrine diseases associate with diabetes and cancer widely happen in people around the world. Stem cell therapeutics and their translational medicine are promising to solve the problems in endocrine diseases. However, there are still essential scientific questions that are not fully understood about stem cells, which hinders their clinical applications. In recent years, research for stem cells and translational medicine has attracted multidisciplinary scientists including cell biologists, chemists, materials scientists, and clinical doctors collaborated to contribute to the innovation. New knowledge and technologies have been found and invented to ensure the safety and curative effect of stem cells for endocrine diseases. This Research Topic provides high-quality research articles and reviews in stem cell and translational medicine research for the treatment and understanding of endocrine diseases.

Diabetes mellitus (DM) is a typical endocrine disease that affects millions of people. Endothelial colony-forming cells (ECFCs) that repair and restore blood vessels have the potential to treat DM patients who require revascularization therapy. Liu et al. reviewed the challenges and clinical prospects of ECFCs for the treatment of diabetic vascular complications. Liu et al. summarized the potential molecular mechanisms for ECFC dysfunction and discussed strategies for enhancing the therapeutic efficacy of ECFCs. Nevertheless, the limitation of ECFCs for clinics in the current stage could be the few results reported in humans. DM is also a complex disease that can be associated with multiple factors. Wang used two-sample Mendelian randomization (TSMR) to analyze the causality between the exposure and the outcome of plasma activin A levels in DM patients. In the conclusion of this study, no causality is found between plasma activin A and DM. Future studies are needed to further confirm the associations in human populations.

Equine metabolic syndrome (EMS) characterized by insulin resistance is an endocrine disease in horses. Elements such as magnesium (Mg), manganese (Mn), and chromium (Cr) are involved in the molecular modulation of insulin sensitivity and liver insulin resistance. Tomal et al. investigated the treatment efficacy of Arthrospira platensis enriched with Cr(III), Mg(II), and Mn(II) ions for EMS-affected horses. This strategy not only reduces baseline insulin and glucose levels but reduces inflammation and obesity-related fat accumulation as well, and therefore has the potential to complement conventional management for EMS.

Stem/Progenitor cells possess multidirectional differentiation potential and secrete various factors for tissue regeneration. However, the therapies of stem cells are limited by insufficient source and mature differentiation. Low-intensity pulsed ultrasound (LIPUS) produces mechanical, thermal, and vibrational stimuli to stem/progenitor cells and their microenvironment. He et al. summarized recent approaches of the LIPUS effect on the biological functions of stem/progenitor cells and discussed the roles and mechanisms of LIPUS in stem/progenitor cells and exosomes. This review provides a perspective on using LIPUS to address the challenges in stem cell-based therapy.

Cytokine Interleukin-6 (IL-6) is reported to promote the occurrence and development of pancreatic cancer, and therefore IL-6 is considered as a biomarker for the diagnosis and prognosis of pancreatic cancer. Song et al. systematically summarized the mechanism of IL-6 inducing the deterioration of pancreatic cells. This review provided knowledge and insight for pancreatic cancer diagnosis and treatment.

Taken together, this Research Topic on Stem Cell and Translational Medicine Research in Endocrine Diseases provides a glimpse of recent innovations in stem cells and the treatment of endocrine diseases. This Research Topic brings together the latest quality articles to explore the latest advancements in stem cell research and translational medicine to understand their impact on endocrine diseases. We hope it facilitates discussions on the potential of stem cell-based therapies, tissue engineering, regenerative medicine, and personalized medicine approaches in addressing endocrine disorders. The interactions between stem cells and the treatment of endocrine diseases should be taken into consideration and investigated in depth. Translational medicine is also needed to help move forward the treatments to the clinic to serve the patients earlier. We hope this Research Topic inspires original ideas for stem cell research, translational medicine, and the treatment of endocrine diseases.

